# Diverse and Atypical Presentations of Acroangiodermatitis of Mali: Diagnostic Insights From Three Cases

**DOI:** 10.7759/cureus.98861

**Published:** 2025-12-09

**Authors:** Greeshma Peddireddy, Divya Raviprakash, Leena Dennis Joseph, Sudha Rangarajan, Adikrishnan Swaminathan

**Affiliations:** 1 Department of Dermatology, Sri Ramachandra Institute of Higher Education and Research, Chennai, IND; 2 Department of General Pathology, Sri Ramachandra Institute of Higher Education and Research, Chennai, IND

**Keywords:** acroangiodermatitis of mali, benign, chronic venous insufficiency, pseudo-kaposi sarcoma, vasoproliferative

## Abstract

Acroangiodermatitis is an uncommon, benign vasoproliferative entity that often develops in the setting of long-standing venous hypertension or arteriovenous abnormalities. Its clinical resemblance to other dermatoses often results in misdiagnosis and delayed treatment. We describe three cases, two males and one female, who presented with persistent lower limb lesions. Clinical findings included hyperpigmented plaques, indurated plaques, and erythematous soft nodules localized to the legs. Clinically, varicosities were evident in two of the three cases, and one patient had previously undergone surgical vein stripping for the same. Given the chronicity of lesions, biopsies were performed. Histopathological features were consistent with acroangiodermatitis, while dermoscopy showed vascular patterns along with white, structureless areas. Venous Doppler ultrasound confirmed the underlying venous insufficiency in all three cases. Based on clinical, dermoscopic, histopathological, and radiological evaluation, a final diagnosis of acroangiodermatitis of Mali was made. The patients were started on oral calcium dobesilate and topical corticosteroids and were referred for vascular surgical evaluation. This case series highlights the importance of recognizing the diverse and atypical presentations of acroangiodermatitis of Mali, emphasizing that biopsy and dermoscopy are critical for a definitive diagnosis.

## Introduction

Acroangiodermatitis (pseudo-Kaposi sarcoma) is an uncommon, benign, vasoproliferative disorder first recognized by Mali et al. in 1965 [1,2]. This condition represents a reactive angiodysplasia of the cutaneous vasculature and is most frequently associated with chronic venous insufficiency, as well as congenital or acquired arteriovenous malformations. Clinically, it appears as red-violaceous or brown macules, papules, nodules, and plaques primarily located on the extensor surfaces of lower limbs [3]. Four variants of acroangiodermatitis have been documented in the literature. The most common variant is the Mali type, which is associated with chronic venous insufficiency and typically presents bilaterally. The second variant, known as Stewart-Bluefarb syndrome, is linked to congenital arteriovenous malformations and presents unilaterally. The third variant occurs in patients with chronic renal failure and those with iatrogenic arteriovenous shunts. The fourth variant is associated with the first pregnancy [1]. Due to its diverse morphology, acroangiodermatitis can resemble benign conditions, such as stasis dermatitis and lichen planus, as well as aggressive malignant conditions, such as Kaposi sarcoma, which can result in diagnostic uncertainty. Therefore, histopathological examination is essential for definitive diagnosis. This case series highlights the diverse and atypical clinical presentations of acroangiodermatitis of Mali and underscores the importance of biopsy in establishing an accurate diagnosis.

## Case presentation

Case 1

**Figure 1:**
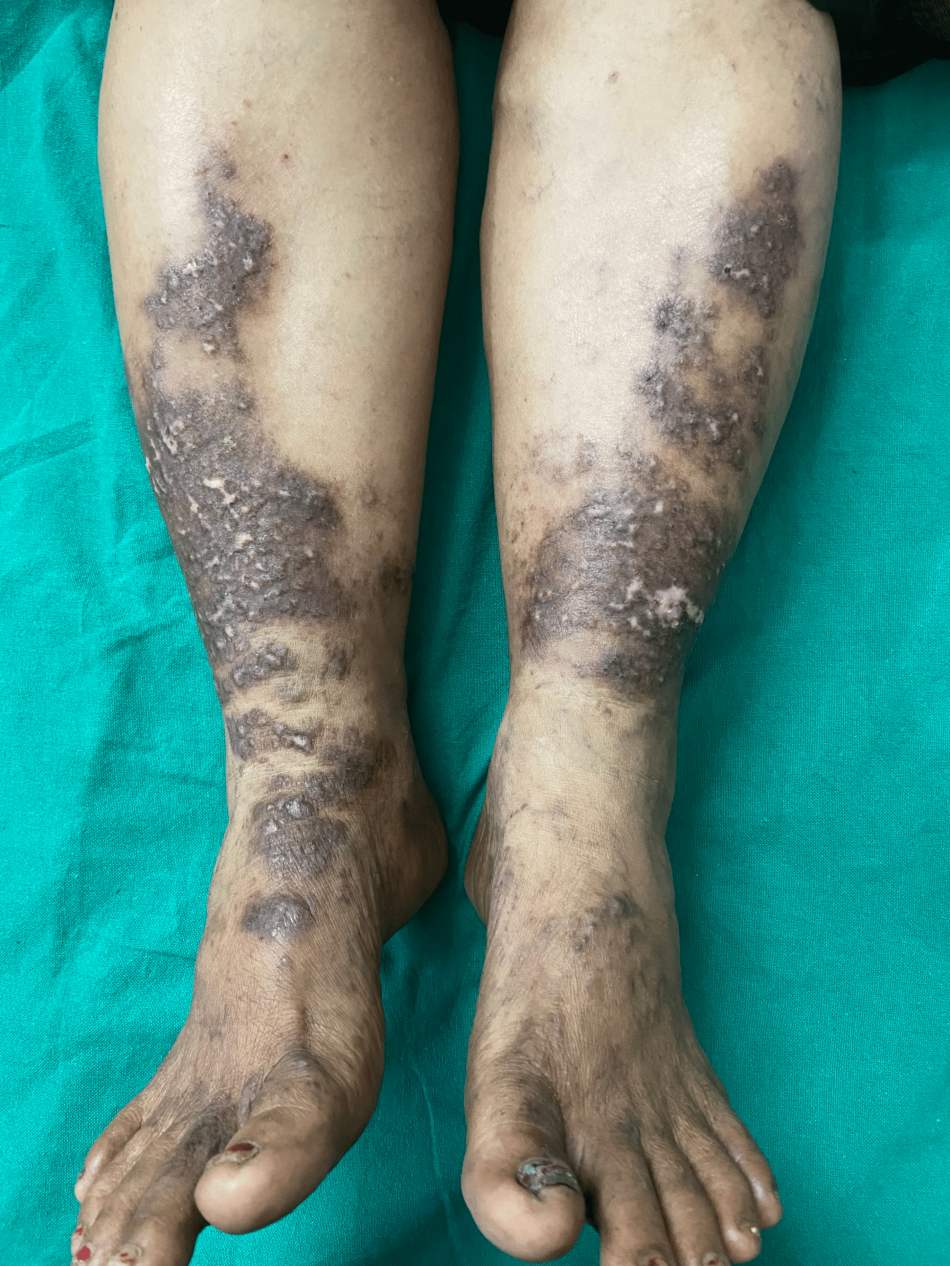
Multiple hyperpigmented plaques studded with papules over bilateral lower limbs with varicosities in the surrounding skin.

A 54-year-old female daily wage worker presented to our outpatient department with multiple dark, raised, itchy skin lesions over both legs. These lesions had gradually progressed in size and number over the past 10 years. The patient had previously been treated for lichen planus with topical corticosteroids, but lesions persisted. On examination, multiple hyperpigmented plaques of sizes ranging from 2 × 1 cm to 15 × 10 cm, studded with papules, were noted over bilateral lower limbs (Figure 1).

**Figure 2:**
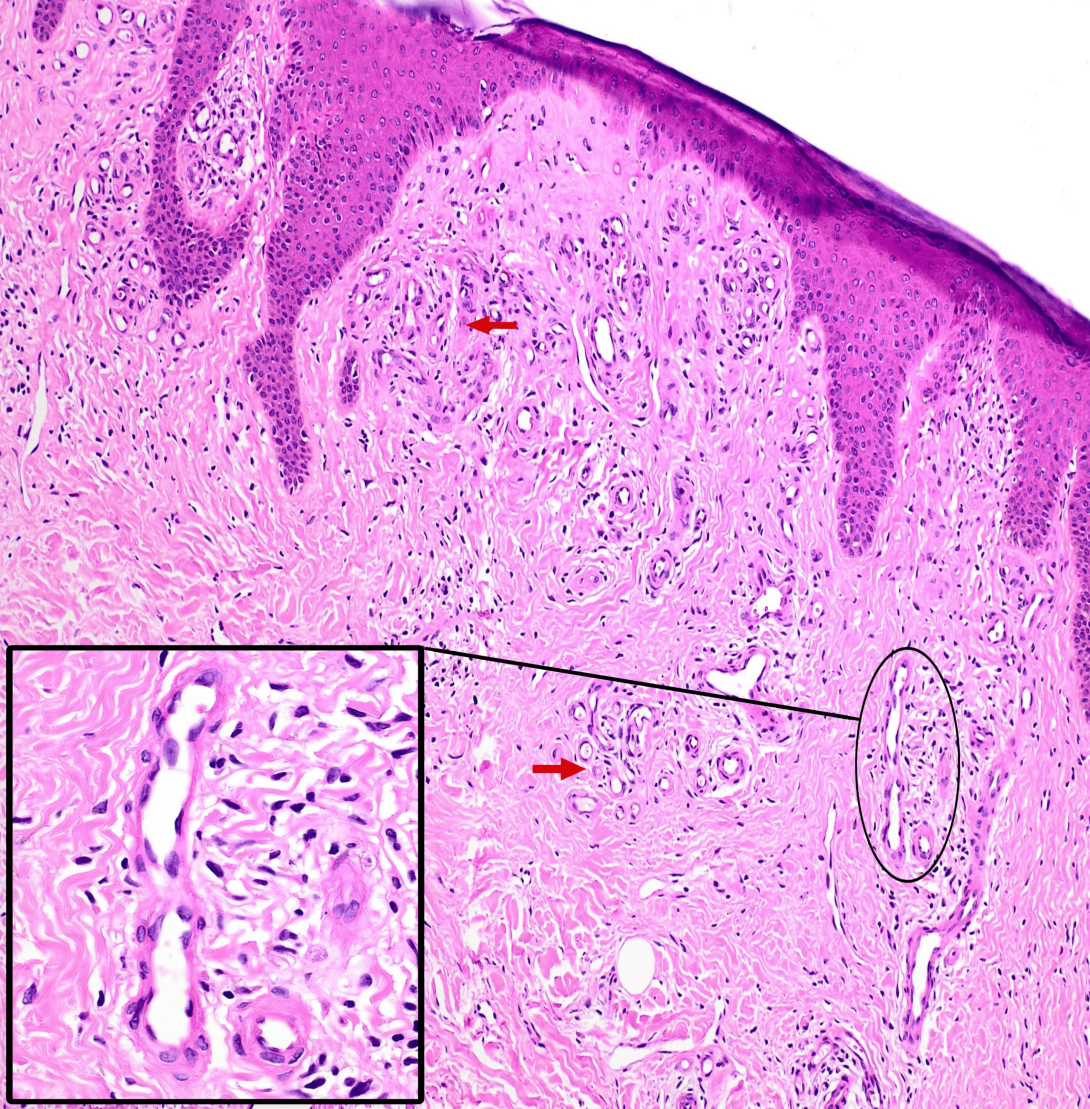
Histopathology showing focal atrophy of the epidermis with flattening of rete ridges, lobules of capillaries (red arrows) with plump endothelial cells (inset) surrounded by a predominantly lymphocytic infiltrate in the papillary dermis (hematoxylin and eosin, ×10).

A few lesions displayed central areas of depigmentation and superficial erosions. Dilated varicosities were noted in the surrounding skin of both lower limbs, and all peripheral pulsations were palpable. There was no evidence of a palpable thrill or local tenderness. The left saphenofemoral junction was found to be incompetent on the Brodie-Trendelenburg test, and bilateral perforator vein incompetence was noted on multiple tourniquet tests. Differential diagnosis included acroangiodermatitis of Mali, prurigo nodularis, and hypertrophic lichen planus. A punch biopsy from a lesion on the right lower leg showed focal atrophy of the epidermis, flattening of rete ridges, and lobules of capillaries with plump endothelial cells surrounded by a predominantly lymphocytic infiltrate in the papillary dermis, suggesting a vascular reactive process (Figure 2).

**Figure 3:**
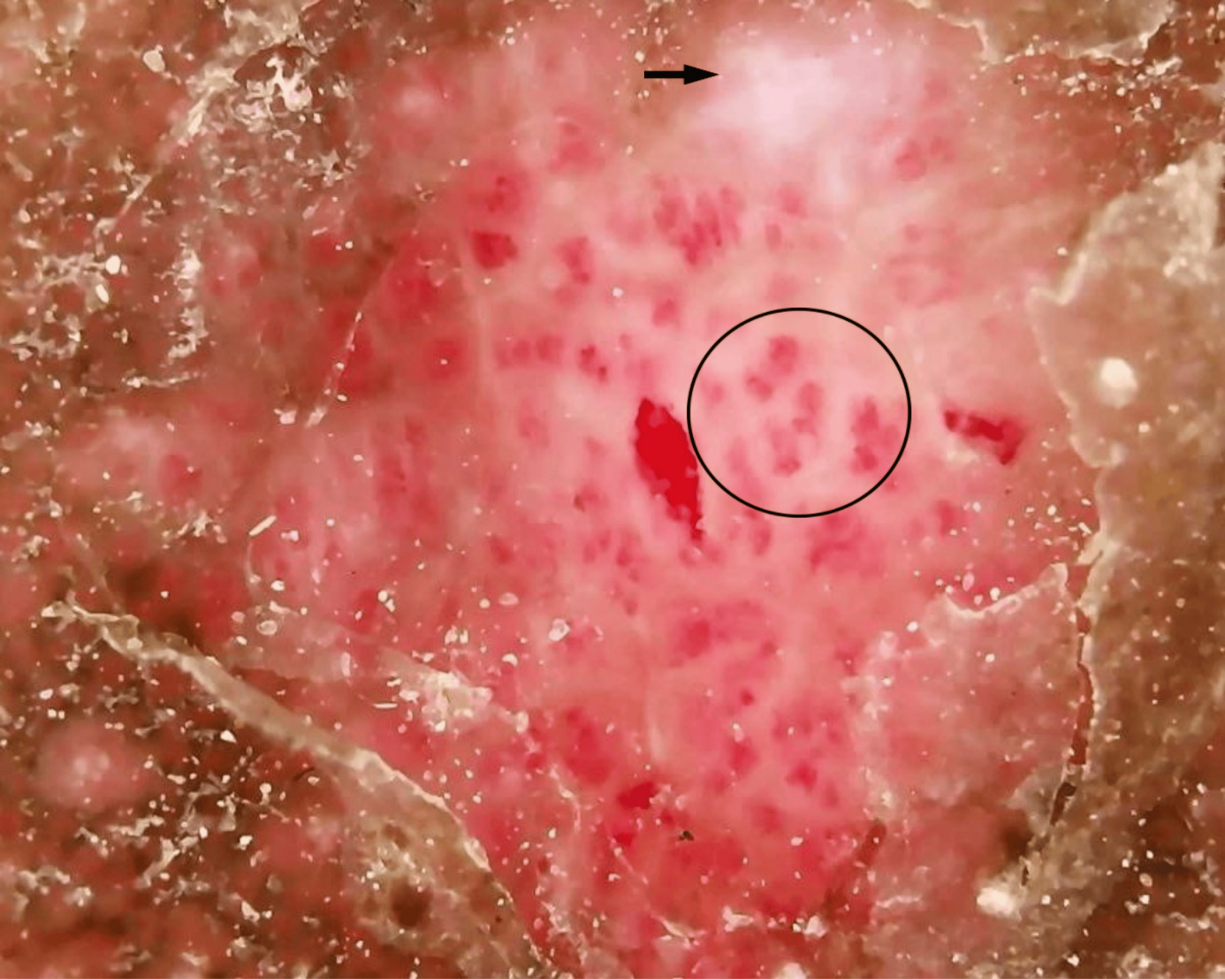
Dermoscopy revealing multiple red dots and globules (black circle) along with white, structureless areas (black arrow).

Dermoscopy revealed multiple red dots and globules suggestive of a vascular reactive pattern, along with white, structureless areas indicative of dermal fibrosis (Figure 3).

Venous Doppler ultrasound of the left lower limb showed incompetence of the saphenofemoral and saphenopopliteal junctions, dilatation of the long saphenous vein and proximal and mid segments of the short saphenous vein, as well as multiple incompetent perforators. The right lower limb venous Doppler also showed dilatation of the long saphenous vein, proximal and mid segments of the short saphenous vein, and three incompetent perforators.

Case 2

**Figure 4:**
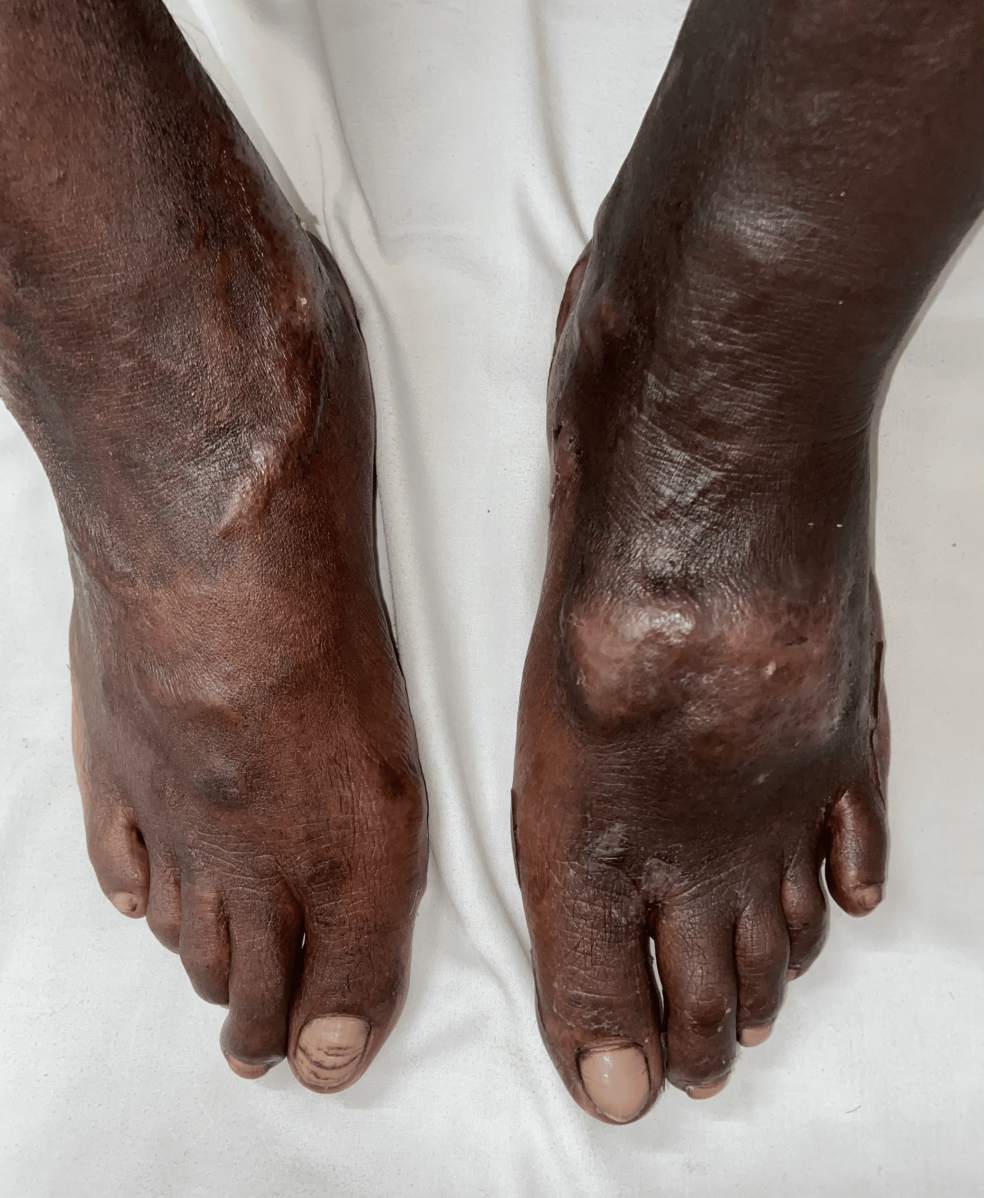
Hyperpigmented to erythematous firm nodules and plaques with surrounding hyperpigmentation over the dorsa of bilateral feet.

A 29-year-old male presented with a 12-year history of recurrent episodes of pain and swelling of both legs, along with asymptomatic dark, raised skin lesions over both legs for the past eight years. He initially noticed a small swelling on the dorsum of his left foot, which gradually progressed to its current size, with lesions extending to the lower third of both legs and dorsa of bilateral feet. There was no family history of similar complaints. The patient had been diagnosed with varicose veins 12 years earlier and underwent surgical vein stripping eight years before presentation. Physical examination revealed multiple firm, hyperpigmented to erythematous nodules and plaques of sizes ranging from 1 × 1 cm to 5 × 4 cm with surrounding hyperpigmentation over the lower third of the bilateral lower limbs and dorsa of bilateral feet (Figure 4).

**Figure 5:**
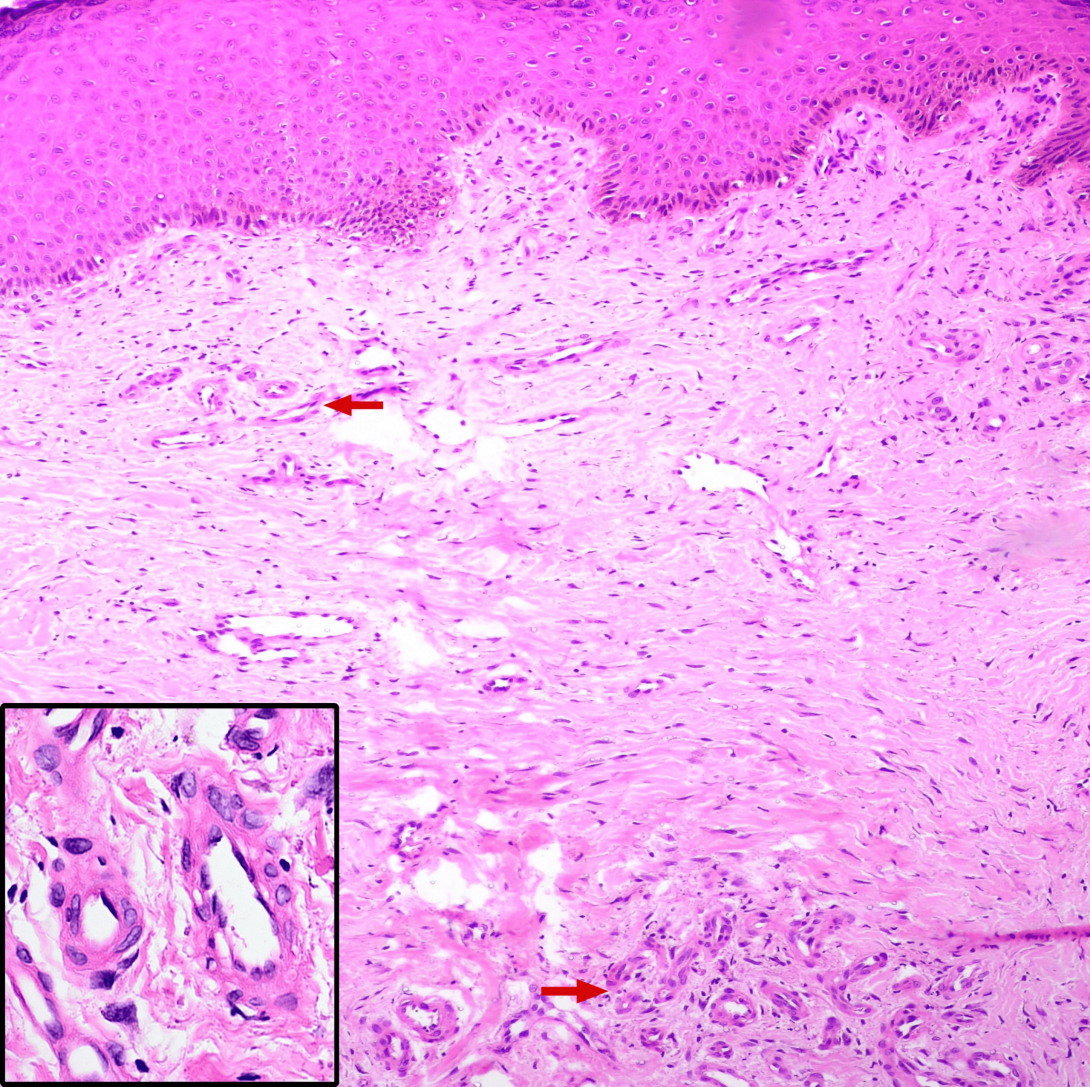
Histopathology showing many congested capillaries (red arrows) lined by plump endothelial cells (inset) along with interstitial infiltrate in the upper and mid dermis (hematoxylin and eosin, ×100).

The peripheral arterial pulsations were palpable, and no local tenderness, audible bruit, or palpable thrill was observed. The saphenofemoral junction in both legs showed incompetence during the Brodie-Tredelenburg test, while the perforators in both legs demonstrated incompetence in multiple tourniquet tests. A differential diagnosis of keloid, deep fungal infections, and acroangiodermatitis of Mali was considered. A lesional skin biopsy showed epidermis with irregular thickening, hyperkeratosis, hypergranulosis, and many congested capillaries with plump endothelial cells, along with interstitial infiltrate in the upper and mid dermis (Figure 5).

**Figure 6:**
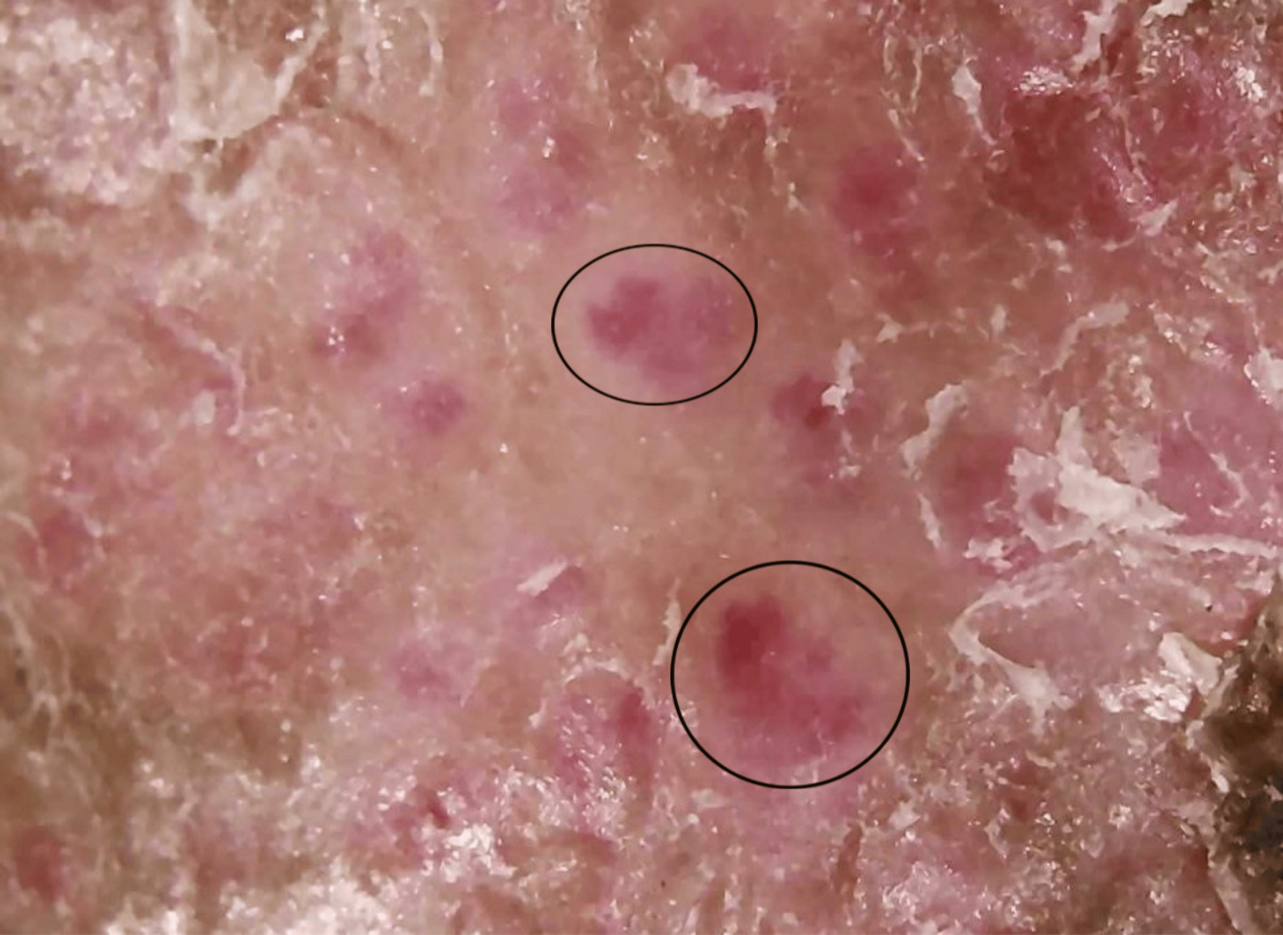
Dermoscopy showing multiple red dots and globules (black circles).

Dermoscopy revealed multiple red dots and globules suggestive of a vascular reactive pattern (Figure 6).

Venous Doppler ultrasound of both the lower limbs demonstrated incompetence at the saphenofemoral and saphenopopliteal junctions, with multiple dilated and incompetent perforators.

Case 3

**Figure 7:**
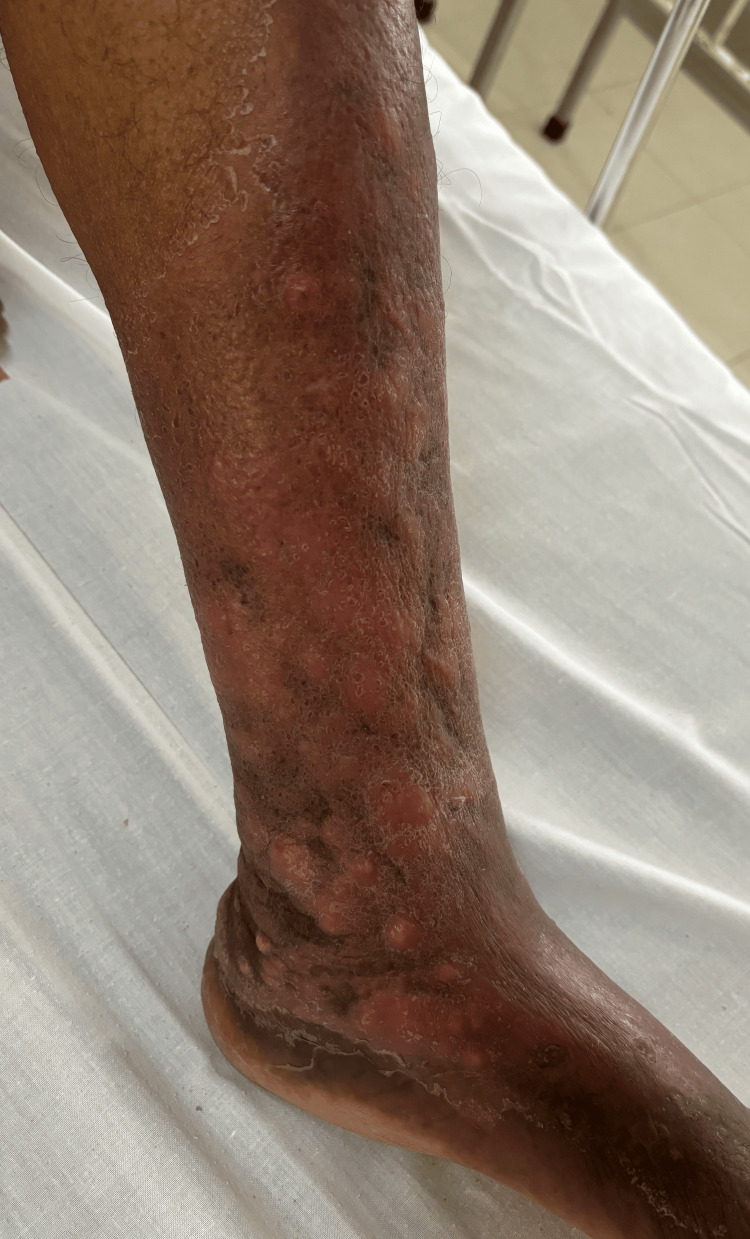
Multiple discrete and coalescing erythematous nodules and plaques extending from below the knee to the dorsum of the left foot.

A 48-year-old farmer presented with pain and swelling in the left leg for one week, along with red, raised lesions over the same leg, which had been gradually progressing in number and size for the past two years. On examination, there were multiple discrete and coalescing soft, compressible erythematous nodules and plaques of sizes ranging from 1 × 1 cm to 10 × 8 cm with surrounding erythema, induration, and peripheral desquamation extending from below the knee to the dorsum of the left foot (Figure 7).

**Figure 8:**
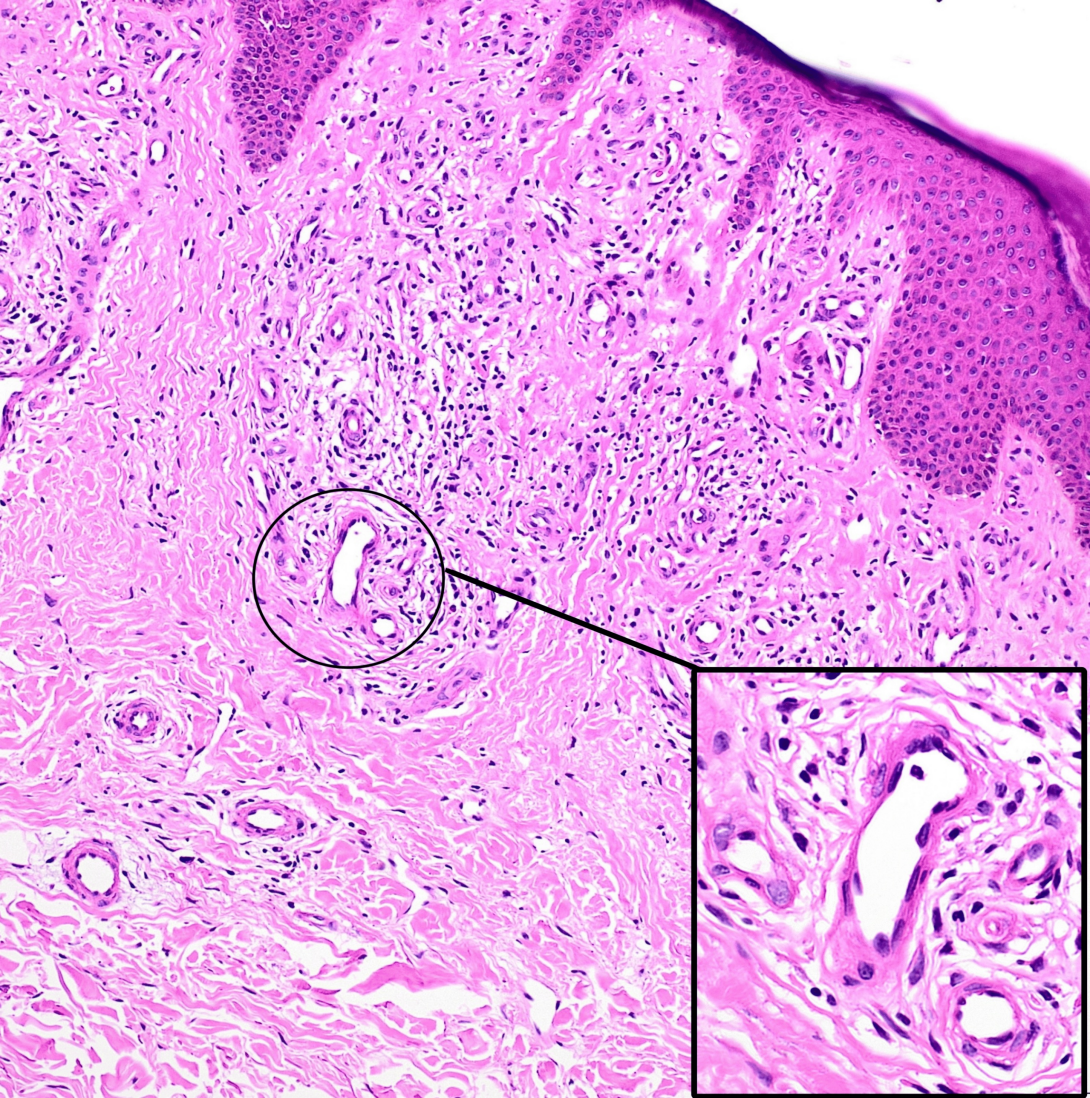
Histopathology showing many blood vessels with plump endothelial cells (inset) surrounded by lymphocytes in the upper and deep dermis (hematoxylin and eosin, 10×).

Localized warmth and tenderness, along with dilated varicose veins in the surrounding skin of the left leg, were noted. No palpable thrill or audible bruit was observed. Examination of the right leg revealed no abnormalities. A differential diagnosis of sporotrichosis, acroangiodermatitis of Mali, and Kaposi sarcoma was considered. A lesional skin biopsy showed epidermis with mild spongiosis, many blood vessels with plump endothelial cells surrounded by lymphocytes, and occasional eosinophils in the upper and deep dermis (Figure 8).

Venous Doppler ultrasound of the left lower limb showed dilated superficial veins and dilated, incompetent perforators.

In all three cases, routine hematological and biochemical investigations were within normal limits, and serological testing for HIV yielded negative results. Based on clinical, histopathological, dermoscopic, and Doppler findings, a final diagnosis of acroangiodermatitis of Mali was established in all three cases.

The first two cases were started on calcium dobesilate at a dosage of 500 mg twice daily, topical emollients, and topical clobetasol propionate 0.05% ointment. Supportive measures, including foot-end elevation and the use of compression stockings, were recommended. Both patients were referred to vascular surgery for further management of varicosities.

The third patient was initially managed for left leg cellulitis with parenteral antibiotics and topical emollients for one week. Following the resolution of cellulitis, treatment was continued with oral calcium dobesilate at 500 mg twice daily and topical clobetasol propionate 0.05% ointment, and compression stockings were advised; however, the patient was lost to further follow-up. Table 1 presents a summary of all three cases.

**Table 1:** Comparative summary of clinical features and diagnostic findings of the three cases in this case series.

Findings	Case 1	Case 2	Case 3
Clinical findings	Multiple hyperpigmented plaques studded with papules over the bilateral lower limbs	Multiple firm, hyperpigmented to erythematous nodules and plaques with surrounding hyperpigmentation over the lower third of the bilateral lower limbs and dorsa of the bilateral feet	Multiple discrete and coalescing soft, compressible erythematous nodules and plaques with surrounding erythema, induration, and peripheral desquamation extending from below the knee to the dorsum of the left foot
Venous examination	The left saphenofemoral junction and bilateral perforator veins demonstrated incompetence	The saphenofemoral junction and perforators in both legs demonstrated incompetence	Not performed
Histopathology	Lobules of capillaries with plump endothelial cells surrounded by a predominantly lymphocytic infiltrate in the papillary dermis	Many congested capillaries with plump endothelial cells, along with interstitial infiltrate in the upper and mid dermis	Many blood vessels with plump endothelial cells surrounded by lymphocytes and occasional eosinophils in the upper and deep dermis
Dermoscopy	Multiple red dots and globules, along with white, structureless areas	Multiple red dots and globules	Dermoscopy was not performed
Venous Doppler findings	Left lower limb: incompetent saphenofemoral and saphenopopliteal junctions, dilated long and short saphenous veins, and multiple incompetent perforators. Right lower limb: dilated long and short saphenous veins and three incompetent perforators	Both the lower limbs demonstrated incompetence at the saphenofemoral and saphenopopliteal junctions, with multiple dilated and incompetent perforators	Left lower limb: dilated superficial veins and dilated, incompetent perforators

## Discussion

Acroangiodermatitis is a reactive angiodysplasia of the pre-existing cutaneous vasculature, which can occur in five distinct clinical settings: chronic venous insufficiency, congenital arteriovenous malformations, acquired iatrogenic arteriovenous fistula in patients with chronic renal failure undergoing hemodialysis, amputation stumps, and paralyzed extremities [3,4]. All three cases presented in our report exhibited chronic venous insufficiency.

The underlying etiology of Mali-type acroangiodermatitis is not fully established. However, it is suggested that chronic venous stasis elevates capillary pressure, leading to chronic edema and tissue hypoxia. This, in turn, results in neovascularization and fibroblast proliferation [4].

Acroangiodermatitis of Mali characteristically appears as purplish papules and plaques, resulting from chronic pressure changes and proliferation of blood vessels in the dermis, along with the extravasation of red blood cells [5].

Variants of acroangiodermatitis include the Mali type, the most common variant of acroangiodermatitis, which typically presents as slowly evolving violaceous or brown macules, papules, nodules, or indurated plaques. This variant predominantly occurs in elderly men and is associated with chronic venous insufficiency. Due to the systemic nature of venous stasis, the lesions are usually bilateral and often symmetrical, affecting the dorsum of the foot, the first and second toes, or the medial aspect of the lower legs [4,6,7]. All of our patients exhibited this variant of acroangiodermatitis. While the first two cases presented bilaterally, the third case was notable for its unilateral presentation, which is considered atypical for mali-type acroangiodermatitis.

Stewart-Blufarb syndrome is typically observed in young adults with an underlying congenital arteriovenous malformation. Clinically, it presents as painful purple macules and papules localized to the lower extremities, most often presenting unilaterally. In some instances, palpable thrill and audible bruit may be detected due to the underlying arteriovenous shunt [4,6,8].

Another variant is acroangiodermatitis occurring in chronic renal failure patients undergoing hemodialysis following the placement of an arteriovenous shunt [4,8]. Finally, acroangiodermatitis occurring in the first pregnancy is known as Dermite ocre of Favre, which presents as gravity purpura on lower legs, dorsa of feet, and toes, mainly over the site of venous varicosities [5].

Clinical differentials include Kaposi sarcoma, lichen planus, stasis dermatitis, angiokeratomas, pigmented purpuric dermatoses, and deep fungal infections such as mycetoma and sporotrichosis [1]. Histopathologic differentials include Kaposi sarcoma, vasculitis, pigmented purpuric dermatitis, and stasis dermatitis [4].

Histopathology serves as the gold standard for distinguishing acroangiodermatitis from Kaposi sarcoma. In acroangiodermatitis, vascular hyperplasia arises from the proliferation of preexisting vasculature, which is accompanied by fibroblastic activity, whereas in Kaposi sarcoma, vascular proliferation is independent of the underlying normal cutaneous vasculature. Proliferating vessels in Kaposi sarcoma exhibit jagged outlines and slit-like inconspicuous lumina, accompanied by endothelial atypia. Conversely, acroangiodermatitis is characterized by a lobular growth pattern of papillary dermal thick-walled, dilated vessels with regular, rounded lumina. These vessels are lined by plump endothelial cells, which show no or minimal atypia. In Kaposi sarcoma, vessels may exhibit the “promontory sign” and have a “back-to-back” appearance, while in acroangiodermatitis, an edematous matrix separates the angiomatous capillaries. In acroangiodermatitis, hyperplastic vessels exhibit CD34 expression and factor VIII-associated antigen on their endothelial cells. In contrast, Kaposi sarcoma shows CD34 expression on both endothelial cells and perivascular spindle cells. Dermal fibrosis, hemosiderin deposition, and red blood cell extravasation are observed in both conditions [3,6,9,10].

In vasculitis, the vessel walls exhibit fibrinoid necrosis with no evidence of vascular proliferation. In stasis dermatitis, the epidermis reveals parakeratosis with spongiosis, and there is more significant dermal involvement with increased hemosiderin deposition when compared to acroangiodermatitis. In pigmented purpuric dermatitis, dilated capillaries are present; however, there is a lack of plump endothelial cell lining and dermal fibrosis [4,10].

Dermoscopic findings of acroangiodermatitis include the presence of irregularly distributed polymorphic vessels and white, structureless areas, which are attributable to vascular proliferation and dermal fibroplasia, respectively [1,11].

Management of acroangiodermatitis involves correction of the underlying vascular abnormality and managing complications. Conservative therapy includes the use of compression stockings, bandages, and pumps, along with venoactive medications such as calcium dobesilate and oxerutin, which can help alleviate symptoms associated with venous stasis. Topical corticosteroid therapy is often beneficial [4,12]. Medical modalities of therapy are limited, which include oral antibiotic regimens such as dapsone 50 mg twice a day for three months or erythromycin 500 mg four times a day for three months [4]. The exact mechanism of action of these agents remains unclear, although erythromycin is believed to exert anti-inflammatory effects by inhibiting chemotaxis of monocytes, leukocytes, and eosinophils [4,13]. Surgical intervention to correct the underlying vascular pathology is the mainstay of treatment for halting the progression of acroangiodermatitis [4,6].

## Conclusions

Acroangiodermatitis is often misdiagnosed due to its relative rarity. Our case series showcases the varied clinical spectrum of acroangiodermatitis, which can often simulate several dermatological conditions, including Kaposi sarcoma, lichen planus, pigmented purpuric dermatitis, angiokeratomas, stasis dermatitis, and deep fungal infections, resulting in diagnostic delay and treatment failure. We present an atypical unilateral case emphasizing that Mali-type acroangiodermatitis should be considered in unilateral presentations. Histopathology is crucial for the early diagnosis of acroangiodermatitis, as timely interventions, such as surgical management of the underlying vascular pathology, are essential. This series underscores the significance of integrating histopathology with dermoscopy to distinguish a benign disorder such as acroangiodermatitis from a more aggressive malignant condition such as Kaposi sarcoma. Addressing the underlying vascular pathology is vital for halting the progression of acroangiodermatitis.
